# Pre- and Early-Postnatal Nutrition Modify Gene and Protein Expressions of Muscle Energy Metabolism Markers and Phospholipid Fatty Acid Composition in a Muscle Type Specific Manner in Sheep

**DOI:** 10.1371/journal.pone.0065452

**Published:** 2013-06-06

**Authors:** Lei Hou, Anna H. Kongsted, Seyed M. Ghoreishi, Tasnim K. Takhtsabzy, Martin Friedrichsen, Lars I. Hellgren, Haja N. Kadarmideen, Allan Vaag, Mette O. Nielsen

**Affiliations:** 1 Department of Veterinary Clinical and Animal Sciences, Faculty of Health and Medical Sciences, University of Copenhagen, Frederiksberg, Denmark; 2 Center for Biological Sequence Analysis, Technical University of Denmark, Lyngby, Denmark; 3 Department of Endocrinology, Rigshospitalet, Copenhagen, Denmark; 4 Department of Nutrition, Exercise and Sports, the August Krogh Centre, University of Copenhagen, Copenhagen, Denmark; 5 Department of Animal Science, University of Jiroft, Jiroft, Iran; 6 Center for Fetal Programming, Copenhagen, Denmark; CRCHUM-Montreal Diabetes Research Center, Canada

## Abstract

We previously reported that undernutrition in late fetal life reduced whole-body insulin sensitivity in adult sheep, irrespective of dietary exposure in early postnatal life. Skeletal muscle may play an important role in control of insulin action. We therefore studied a range of putative key muscle determinants of insulin signalling in two types of skeletal muscles (*longissimus dorsi* (LD) and *biceps femoris* (BF)) and in the cardiac muscle (*ventriculus sinister cordis* (VSC)) of sheep from the same experiment. Twin-bearing ewes were fed either 100% (NORM) or 50% (LOW) of their energy and protein requirements during the last trimester of gestation. From day-3 postpartum to 6-months of age (around puberty), twin offspring received a high-carbohydrate-high-fat (HCHF) or a moderate-conventional (CONV) diet, whereafter all males were slaughtered. Females were subsequently raised on a moderate diet and slaughtered at 2-years of age (young adults). The only long-term consequences of fetal undernutrition observed in adult offspring were lower expressions of the insulin responsive glucose transporter 4 (*GLUT4*) protein and peroxisome proliferator-activated receptor gamma, coactivator 1α (*PGC1α*) mRNA in BF, but increased PGC1α expression in VSC. Interestingly, the HCHF diet in early postnatal life was associated with somewhat paradoxically increased expressions in LD of a range of genes (but not proteins) related to glucose uptake, insulin signalling and fatty acid oxidation. Except for fatty acid oxidation genes, these changes persisted into adulthood. No persistent expression changes were observed in BF and VSC. The HCHF diet increased phospholipid ratios of n-6/n-3 polyunsaturated fatty acids in all muscles, even in adults fed identical diets for 1½ years. In conclusion, early postnatal, but not late gestation, nutrition had long-term consequences for a number of determinants of insulin action and metabolism in LD. Tissues other than muscle may account for reduced whole body insulin sensitivity in adult LOW sheep.

## Introduction

A rapid burst of fetal growth takes place during late-gestation, and maternal nutritional restriction during this period can reduce fetal growth rate and result in low birth weight (LBW) of the new-borns [1], [2]. It is well-known that LBW is associated with increased risk of type II diabetes, obesity, and other metabolic disorders later in life [3], [4], [5], [6]. However, it is not fully understood, to what extent different dietary combinations in pre– and postnatal life can contribute to the development of whole-body insulin resistance in LBW individuals [4], [7], [8].

The molecular-biological mechanisms which are linking a LBW phenotype to increased risk of developing metabolic disorders in later life, are only partially understood [9]. Both human and rat studies have shown that LBW is associated with distinct changes in protein expression patterns with down-regulation of key markers of skeletal muscle insulin sensitivity, such as glucose transporter 4 (*GLUT4*), phosphatidylinositol 3-kinases (*PI3K*s) and protein kinase C zeta (*PKCζ*) [10]. In addition, decreased transcription of the nuclear transcription factor peroxisome proliferator-activated receptor gamma, coactivator 1 alpha (*PGC1α*) has been associated with muscle insulin resistance in patients with overt type II diabetes [11], in LBW twins [12] and in short-term over-fed LBW singletons [13]. Muscle is therefore believed to be a key target organ for fetal programming, which contributes to the development of whole-body insulin resistance later in life in LBW subjects.

In addition to transcriptional and protein expression pathways, modifications of lipid content and phospholipid (PL) fatty acid (FA) composition are also believed to play a role in the development of muscle insulin resistance. Increased intramyocellular lipid content, and the often associated mitochondrial oxidative stress, are highly linked to skeletal muscle insulin resistance [14], [15], [16]. Furthermore, insulin sensitivity in muscle has also been reported to be correlated to the content of omega-3 polyunsaturated fatty acids (n-3 PUFA) in skeletal muscle PL [17], [18]. It has, however, not been clarified, if this association between muscle insulin sensitivity with muscle lipid content or FA-composition in fact reflect direct cause-and-effect relationships.

The choice of animal model is an important issue when muscle development during the late-gestation period is in focus. Compared to the rat, where secondary myogenesis continues in the postnatal period, fetal muscular development in sheep is more comparable to that of humans, where secondary myotube formation is completed before entering the third trimester [19], [20], [21]. We have previously reported from our Copenhagen sheep model that offspring exposed to undernutrition during late gestation (LOW) developed insulin insensitivity in postnatal life, whereas exposure in early postnatal life to a high-carbohydrate-high-fat (HCHF) diet impaired the ability to secrete insulin. When combining the prenatal LOW and postnatal HCHF nutritional exposures, clear indications of glucose intolerance were observed. [22], [23]. We hypothesised that muscle glucose-insulin signalling and oxidative pathways as well as muscle lipidomes are key targets for and/or determinants of fetal programming induced by late-gestation undernutrition, which in turn predisposes for insulin insensitivity later in life. We also hypothesised that early-postnatal over-nutrition can modify the phenotypic manifestations of the fetal programming induced in late-gestation. To dissect the differential contributions of different muscle types in the development of whole-body insulin insensitivity and glucose intolerance [24], [25], [26], three distinct muscle types were investigated in our sheep model, namely two skeletal muscles, *biceps femoris* (BF, type I fibre dominating, oxidative) and *longissimus dorsi* (LD, type II fibre dominating, glycolytic); and one cardiac muscle, *ventriculus sinister cordis* (VSC).

## Materials and Methods

### Experimental Animals and Treatments

All the experimental animal handling protocols were approved by The Danish National Committee on Animal Experimentation. The animal experiments were conducted at the experimental farm Rørrendegård of the Faculty of Health and Medical Sciences, University of Copenhagen, Denmark. Detailed information about the Copenhagen sheep model and experimental procedures have been reported previously [23]. An overview of the experimental 2×2 factorial design is shown in Information S1. In short, 40 Shropshire lambs from 20 multiparous twin-bearing dams were housed indoors in individual pens. Four lambs died before they reached 6-months of age, and the remaining 36 lambs were included in the present experiment. During the last six weeks of gestation, equivalent to the last trimester (term = 147 days), ten of the dams were fed a diet fulfilling their requirements for energy and protein (NORM), whereas the other ten ewes were fed restricted amounts of the same diet corresponding to only 50% of the NORM diet (LOW). Three days after birth, the dams were removed and the lambs were housed in individual pens. From 3-days until 6-months of age, one lamb from each twin pair received a conventional diet (CONV) consisting of milk replacer (until weaning at 8-weeks of age) and artificially dried green hay. Amounts were adjusted weekly to achieve constant and moderate daily growth rates throughout the differential feeding period (250 g/day). The other twin lamb was assigned to a high-carbohydrate-high-fat diet (HCHF) consisting of popped maize (max. 1 kg/day), dairy cream (38% fat; max. 0.5 L/day), and milk replacer (max. 2.0 L/day during the first eight weeks and 0.5 L/day from 8-weeks to 6-months of age). This 2×2 factorial design resulted in four experimental groups: NORM/CONV, NORM/HCHF, LOW/CONV, and LOW/HCHF. All lambs had free access to water and a vitamin-mineral mix. At 6-months of age, half of the lambs were slaughtered from each treatment group (around puberty, lambs; n = 17, including 14 males and 3 females; all females were in the NORM/CONV group; see Information S1). The rest of the lambs (exclusively females) were managed as one flock and fed a moderate grass-based diet, until they were slaughtered at 2-years of age (young adulthood, sheep; n = 19, all of them were females; see Information S1). After slaughtering, biopsies from BF, LD, and VSC muscles were quickly dissected out, weighed, and snap frozen in liquid nitrogen. Samples were kept at −80°C until further analyses.

### Protein Extraction and Western Blotting

Protein lysate was prepared as described by Friedrichsen *et al.* [27]. All chemicals were obtained from Sigma-Aldrich (Brøndby, Denmark). Total protein content was measured by the bicinchoninic acid assay according to the manufacturers recommended procedures (Thermo Fisher Scientific, Waltham, MA, USA).

To determine protein expression, western blotting was performed as recommended by the manufacturer (Invitrogen, Carlsbad, CA, USA). In short, 20 µg of muscle protein from each sample was separated on 10% Bis-Tris gels (Invitrogen), and blotted on nitrocellulose membranes (Invitrogen). As loading control we used β-tubulin (Cell Signalling Technology, Danvers, MA, USA). However, the protein expression of β-tubulin was unstable and hence not suitable for loading control (Information S2). Therefore, we applied another method as described by Romero-Calvo *et al.* [28]. The total protein content on the membranes were determined as the colour intensity of the Ponceau S stain between 37.1–82.2 kDa (according to Bench Mark pre-stained protein ladder from Invitrogen). After the Ponceau S staining, the membranes were divided at 60 kDa to facilitate incubation with multiple antibodies. Membranes were blocked in 5% skimmed milk prior to incubation with other antibodies and subsequently incubated overnight with the antibodies against *PKCζ* (SC-216, Santa Cruz, CA, USA; see Information S3, S5, and S6 for band specificity and quality), insulin receptor beta subunit (*INSRβ*)(SC-711, Santa Cruz; see Information S3, S5, and S6 for band specificity and quality), phosphoinositide 3 kinase-p85 regulatory subunit (*PI3K-p85*)(06–195, Millipore, Billerica, MA, USA; see Information S4 for band specificity and quality), and phosphoinositide 3 kinase-p110 catabolic subunit (*PI3K-p110*)(SC-602, Santa Cruz; see Information S4 for band specificity and quality), or were incubated with the antibody against *GLUT4* (ab37445, AbCam, Cambridge, UK; see Information S3, S5, and S6 for band specificity and quality). Subsequently, membranes were incubated with horseradish peroxidase-conjugated anti-rabbit antibodies (#7074, Cell Signalling Technology). The signal was detected with LumiGLO reagent (Cell Signalling Technology), and the staining was visualized with a LAS-3000 Image-reader (Fujifilm, Tokyo, Japan), and quantified by MultiGauge version 2.0 software (Fujifilm). Protein contents were expressed in arbitrary units, which were relative to a sheep muscle standard-sample that was loaded in duplicates on each gel (intra-assay variation <10% for each protein analyzed). Assay linearity between signal and protein quantity was verified for each antibody and for the Ponceau S staining. The standards were loaded to wells at the same position of each gel, and the samples were randomly loaded to wells at the rest of the positions.

### RNA Extraction and cDNA Synthesis

Total mRNA was extracted by the following procedures: About 30 mg of muscle biopsy was homogenized (TissueLyser II, QIAGEN, Hilden, Germany) in TRI Reagent (Sigma-Aldrich). The Sigma-Aldrich’s protocol for TRI Reagent was followed, until the colourless upper aqueous phase was acquired through the phase separation step using 1-Bromo-3-Chloropropane (Sigma-Aldrich). This aqueous phase was transferred to a fresh Eppendorf tube, mixed with 500 µl isopropanol (Sigma-Aldrich) by vortexing, and then incubated at −20°C overnight. Afterwards, the supernatant was transferred to SV Total RNA Isolation System (Promega, Madison, WI, USA) for further RNA extraction, according to the protocol provided by Promega. Quantity and concentration of isolated RNA were determined by measuring its absorbance on Nanodrop (Thermo Fisher Scientific, Waltham, MA, USA), and integrity of isolated RNA was confirmed by Agilent 2100 bioanalyzer (Agilent Technologies, Santa Clara, CA, USA) with Agilent RNA 6000 Nano Kit (Agilent Technologies). RNA samples were used for cDNA synthesis only when RIN values were ≥6.5.

For cDNA synthesis of each sample, 1 µg of total RNA was reverse transcribed (RT). The RT reaction master mix was made up of 1 µg total RNA, 5 µl M-MLV 5 x Reaction Buffer (Promega), 1.3 µl dNTP Mix (Promega), 0.2 µl Random Primers (Promega), 0.4 µl Oligo(dT)_15_ Primer (Promega), 0.8 µl RNasin Ribonuclease Inhibitor (Promega), and 1 µl M-MLV reverse transcriptase (Promega). The total reaction volume was made up to 25 µl using nuclease-free water (Promega). For each RT reaction, negative controls (no mRNA template or no reverse transcriptase) were included to ensure that there was no genomic DNA contamination. All cDNA samples were stored at −80°C until further analysis.

### Quantitative Real-time PCR (qPCR)

The mRNA expression levels of target genes in muscles were determined by qPCR. For each gene, a six-point standard curve was made for each muscle type. The efficiencies of primers were between 1.8 and 2.1 (this equals an increase between 80% and 110% of target nucleic acid in each amplification cycle) (Table 1), and all coefficients of determination ≥0.99. LightCycler 480 SYBR Green I Master (Roche Applied Science, Penzberg, Germany), LightCycler 480 Multiwell Plate 384, clear (Roche Applied Science), and LightCycler 480 instrument (Roche Applied Science) were used for qPCR. The reaction volume of each well was 10 µl, which contained 2 µl ten-times diluted cDNA, 5 µl 2 × SYBR Green I master mix (Roche Applied Science), 1 µl 10 µM forward primer (TAG Copenhagen, Copenhagen, Denmark), 1 µl 10 µM reverse primer (TAG Copenhagen), and 1 µl nuclease-free water (Roche Applied Science). Samples and negative controls (no cDNA template) were run in triplicates. The program of each qPCR amplification cycle included: denaturation at 95°C for 30 sec, annealing at 60°C for 30 sec, elongation at 72°C for 45 sec; this cycle was repeated 40 times in each qPCR reaction. Melting curves of PCR products were checked by LightCycler 480 instrument ver. 2.0 software (Roche Applied Science) to ensure uniformity of the PCR-product. Data were analyzed using the advanced relative quantification method provided by the LightCycler 480 instrument ver. 2.0 software. Beta-actin was used as the reference gene to normalize expression levels of target genes. The original source of published primer sequences are listed in Table 1. The primer pair for insulin receptor substrate 1 (*IRS1*) was designed by Primer3 ver. 0.40 (http://frodo.wi.mit.edu/primer3/input.htm) [29]. The primer sequences for *IRS1* were forward 5′-cac ttc gcc tac cat ttc c-3′ and reverse 5′-tgc att tcc aga ccc tcct-3′. The primer sequences of carnitine palmitoyl transferase I (*CPT1*), leptin, and peroxisome proliferator-activated receptor gamma, coactivator beta isoform (*PGC1β*) were provided by Dr. Jacob B. Hansen, Department of Biomedical Sciences, Faculty of Health and Medical Sciences, University of Copenhagen. Primer sequences of *CPT1* were forward 5′-gatctatctgtctggggtcagg-3′ and reverse 5′-ccacgttgtagcaggagga-3′; primer sequences of *leptin* were 5′-cctctcctgagtttgtccaag-3′ and reverse 5′-gcgaggatctgttggtagatt-3′; and primer sequences of PGC1*β* were 5′-cagtggtgcccagagaact-3′ and reverse 5′-agggcctcattctcactgtc-3′.

**Table 1:** References and efficiencies of the primer sequences that were used for qPCR.

Genes and Accession Number in NCBI GenBank (reference)	Efficiency^a^		
	BF	LD	VSC
**Insulin receptor-beta subunit** *(INSRβ*, Y16092) [52]	1.90	1.92	1.85
**Insulin receptor substrate** *1 (IRS1*, XM_581382.3)	1.86	1.80	1.82
**Glucose transporter** **4** (*GLUT4*, AB005283.1) [24]	1.98	2.04	1.84
**Carnitine palmitoyltransferase** I (*CPT1*, NM_001009259)	2.06	2.07	ND
**Uncoupling protein** **3** (*UCP3*, unknown) [53]	2.02	1.86	1.98
**Peroxisome proliferator-activated receptor alpha** (*PPARα*, AY369138) [54]	1.89	2.00	1.94
**Peroxisome proliferator-activated receptor gamma** (*PPARγ*, NM_001100921) [24]	2.01	1.83	1.91
**Peroxisome proliferator-activated receptor delta** (*PPARδ*, unknown) [53]	1.98	2.01	1.89
**Peroxisome proliferator-activated receptor gamma coactivator alpha isoform** (*PGC1α*, NM_177945) [24]	1.91	1.99	1.91
**Peroxisome proliferator-activated receptor gamma coactivator 1 beta isoform** (*PGC1β*, XR_027478)	2.06	1.91	2.06
**Leptin** (U84247)	2.10	2.10	ND
**Fat mass and obesity-associated protein** (*FTO*, EU072419.1) [55]	2.02	2.00	ND
**Glucose transporter 1** (*GLUT1*, U89029.1) [24]	ND	ND	1.90
**AMP-activated protein kinase alpha-2** (*AMPKα2*, NM_214266) [24]	ND	ND	1.96
**Voltage-dependent anion-selective channel protein 1** (*VDAC1*, NM_001126352) [24]	ND	ND	1.88
**Fatty acid binding protein 3** (*FABP3*, NM_174313) [24]	ND	ND	1.98
**Alpha-1 andrenegic receptor** (*ADRα1*, NM_174498) [24]	ND	ND	1.95
**Beta-1 andrenegic receptor** (*ADRβ1*, NM_194266) [24]	ND	ND	1.81
**Beta-2 andrenegic receptor** (*ADRβ2*, NM_174231) [24]	ND	ND	2.00
**Glucocorticoid receptor** (*GR*, X70407.1) [24]	ND	ND	1.97
**Beta-actin** (*ACTB*, AY141970) [56]	1.97	1.90	1.97

The three muscles were: *biceps femoris* (BF), *longissimus dorsi* (LD), and *ventriculus sinister cordis* (VSC).

ND = not determined.

a Efficiency = 10^−1/(slope of standard curve)^. Efficiency was calculated by LightCycler software (ver 2.0, Roche Applied Science, Penzberg, Germany).

### Lipid and Sphingolipid Analysis

Total lipid were extracted from the muscle samples according to the Folch procedure, with addition of internal standards to the chloroform:methanol (Merck KGaA, Darmstadt, Germany) mixture prior to extraction [30]. The internal standards were glyceryl trinonadecanoate (Sigma-Aldrich), nonadecanoic acid, glycerophoshorylcholine di-nonadecanoyl (Larodan, Limhamn, Sweden), and ceramide with C-17 long-chained base (Avanti Polar Lipids, Alabaster, AL, USA) was added to the extraction medium (Merck KGaA, Darmstadt, Germany). PL and triglyceride (TG) were analysed as described earlier [31]. In brief, PL and TG were isolated using thin layer chromatography, visualized with 2,7-dichlorofluoresceine, scraped into glass tubes, and trans-methylated, and analysed using GLC-FID as described by Drachmann et al [31]. Peaks were identified using authentic standards, and quantification was based on peak areas of the internal standards. Ceramide (Cer) analysis was performed as earlier described [31], with the exception that Cer content was quantified using the above-mentioned ceramide C-17 as internal standard.

### Statistical Analysis

Statistical tests on qPCR and western blotting data were performed in SAS software version 9.2 (SAS Institute, Cary, NC, USA); qPCR data were logarithm-transformed to fit normal distribution. The data sets were analysed by Proc Mixed models, which included maternal diet, postnatal diet, age, and their interactions as fixed effects, and the interaction of ewe and lamb as the random effect; derived from the combinations of maternal diet, postnatal diet and age, eight classes were formed (i.e. 6moNORM/CONV, 6moNORM/HCHF, 6moLOW/CONV, and 6moLOW/HCHF; 2yrNORM/CONV, 2yrNORM/HCHF, 2yrLOW/CONV, and 2yrLOW/HCHF). The qPCR and western blotting results were presented as least square mean ± standard error of the mean. Muscle lipidome data were analysed by two-way ANOVA with Prism 5 software (GraphPad Software, San Diego, CA, USA), and results were presented as mean ± standard deviation. Data for lipid FA-composition were analyzed by principal component analysis (PCA) using the LatentiX ver 1.0 Software Package (Latent5, Copenhagen, Denmark, http://www.latentix.com,), and graphs were generated from Prism 5 software (GraphPad Software, USA). *P*<0.05 was considered significant for all analysis.

### Protein-protein Interaction (PPI) Networks Analyses for Candidate Genes

The 22 candidate genes studied here are known gene targets/biomarkers of muscle energy metabolism. It could be hypothesised that if the pre- and postnatal nutritional insults can modify the expression levels of these markers, it would also affect the genes/markers that are very closely co-regulated along with our target genes. This would result in corresponding changes in proteins/metabolite abundance in different tissues. In order to study the regulatory and interaction networks including our target genes (in Table 1), we built PPI networks by anchoring target genes and derived known and predicted interactions by running the network analyses. It was implemented in STRING (Search Tool for the Retrieval of Interacting Genes/Proteins; http://string.embl.de/) [32], which is a database of known and predicted protein interactions. STRING uses one protein per gene. If there is more than one isoform per gene, the longest isoform is selected, unless there is information to suggest that another isoform is better annotated. The interactions include direct (physical) and indirect (functional) associations; they are derived from four sources: Genomic context, High-throughput experiments, Coexpression databases and previous knowledge (text mining).

## Results

There were no significant interactions between pre- and postnatal nutrition exposures for any of the studied parameters unless explicitly stated.

### Birth Weight of Lambs, and Phenotypic Manifestation of Glucose-insulin Axis Function in Adolescent Lambs and Young Adult Sheep

Results for whole-body insulin sensitivity and glucose tolerance in the experimental animals have been reported elsewhere [22]. A summary of the main findings are given here, as it is considered necessary to allow interpretation of the muscle related parameters reported in the present study. In short, the maternal LOW diet significantly reduced the birth weight of female lambs, and tended to reduce the birth weight of male lambs [23]. At 6-months of age, the HCHF lambs had become obese (>30% fat in soft tissue), and had impaired glucose-stimulated secretion of insulin. However, they were still normoglucotolerant indicative of compensatory mechanisms in peripheral target tissues to increase insulin sensitivity and normalize glucose uptake. Clearance of exogenous insulin was also reduced in the HCHF lambs during the initial phase after an intra-venous bolus injection. The prenatally undernourished LOW lambs, however, had increased glucose-stimulated pancreatic insulin secretion without any improvement of glucose clearance, indicating reduced peripheral insulin sensitivity. If LOW lambs were exposed to the mismatching HCHF diet (LOW/HCHF), glucose tolerance developed, indicating that the HCHF diet repressed the compensatory up-regulation of pancreatic insulin secretion observed in the LOW/CONV animals. There were no gender specific effects for any of those indicators of glucose-insulin axis function in the 6-months old animals, where both male and female lambs were studied. The detrimental effects of the HCHF diet on glucose-insulin axis function could no longer be visualized in the young adult sheep (only females were studied at this age) after they had been fed a moderate (and for HCHF sheep: obesity correcting) diet for 1.5-years. Negative impacts of a history of prenatal under-nutrition, however, continued to be evident in the adult LOW ewes, as they cleared exogenously administrated insulin less efficiently compared to the NORM ewes.

In the following it must be born in mind that gender specific effects could not be evaluated for the muscle specific parameters studied, since muscle samples from slaughtered 6-months old animals were derived from primarily males, and samples from 2-year old animals were derived from exclusively females.

### Protein Expressions of Insulin Signalling Molecules in BF, LD, and VSC Muscles

Protein expressions of *INSRβ*, *PKCζ*, and *GLUT4* were measured in all the three muscle types. In VSC muscle the protein expressions of *PI3K-p85* and *PI3K-p110* were also measured.

**Figure 1:**
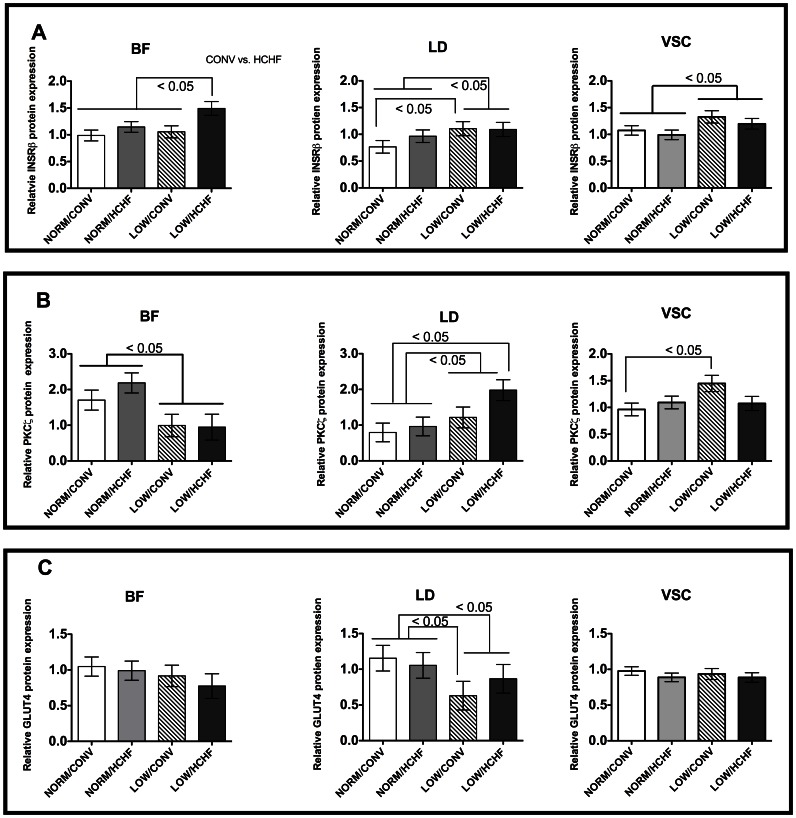
Protein expressions of insulin signalling molecules in muscles of 6-months old lambs. The three muscles were: *biceps femoris* (BF), *longissimus dorsi* (LD), and *ventriculus sinister cordis* (VSC). Panel A, insulin receptor-β subunit (*INSRβ*); panel B, protein kinase zeta (*PKCζ*); panel C, glucose transporter 4 (*GLUT4*). Muscle types are labelled on top of each graph. Data are the ratios of sample to the loading control for each target. Data are expressed as least square mean ± standard error of the mean. Experimental factors with P-values <0.05 are shown at the top-right corner for each graph. NORM/CONV, NORM/HCHF, LOW/CONV, LOW/HCHF refer to experimental treatment groups. NORM and LOW refer to the prenatal nutrition offered to the twin-pregnant dams and fulfilling 100% and 50%, respectively, of daily requirements for energy and protein. CONV and HCHF refer to a moderate or high-carbohydrate-high-fat diet, respectively, fed during the first 6 months of postnatal life.

We observed clear effects of the prenatal nutrition on expression of all the studied proteins in the 6-months old lambs (predominantly males), but the consequences of exposure to a LOW level of nutrition during prenatal life were differentially manifested in the different muscle types (Figure 1):

*INSRβ* protein expression (Figure 1A) was increased in LOW lambs in all the three muscle types (BF, *P = *0.01; LD, *P = *0.03; VSC, *P = *0.03) (Figure 1A). In BF, this could be ascribed to high *INSRβ* protein expression in the LOW/HCHF lambs.

*PKCζ* protein expression (Figure 1B) was reduced in LOW lambs in BF (*P = *0.04), increased in LD (*P = *0.02), and it was also increased in VSC, but only in the LOW/CONV lambs (LOW/CONV vs. NORM/CONV, *P = *0.02).

*GLUT4* protein expression (Figure 1C) was reduced in LD (*P = *0.02) in LOW lambs.

*PI3K-p85* protein expression was reduced by 10% in VSC of LOW compared to NORM lambs (*P = *0.01), while that of *PI3K-p110* was unaffected by the prenatal diet (data not shown).

A long-term effect of exposure to the prenatal LOW level of nutrition was observed only for *GLUT4*, where protein expression in BF was significantly reduced in 2-years old LOW compared to NORM sheep (*P* = 0.05), when the data sets from the 6-months old lambs and the 2-years old sheep were combined (data not shown).

The HCHF diet provided in early postnatal life had no clear effect on protein expressions in neither the male lambs nor in the adult females.

### The mRNA Expressions of Target Genes in BF, LD, and VSC Muscles

**Figure 2:**
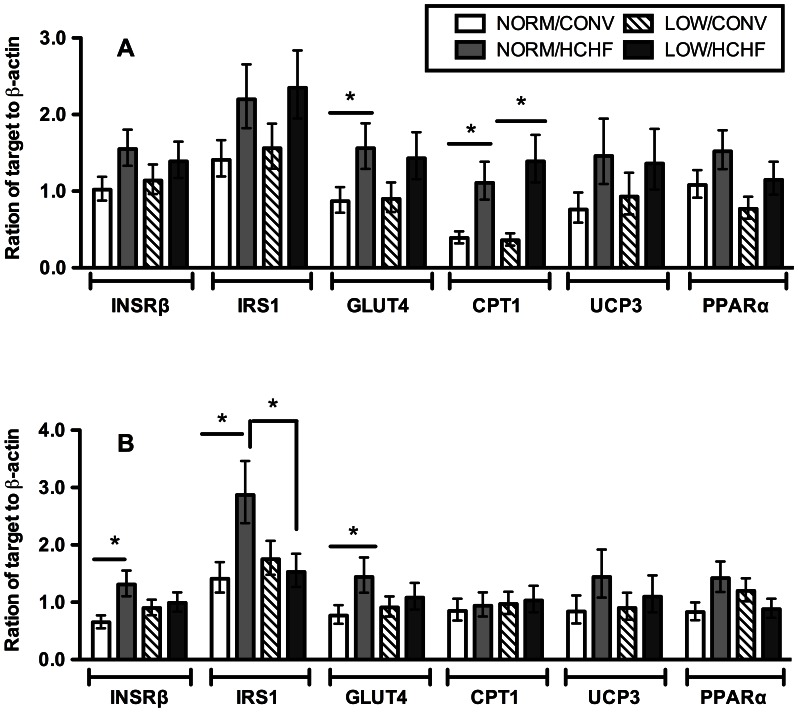
mRNA expressions of selected genes in *longissimus dorsi* muscle of 6-months and 2-years old progenies. The mRNA expression of the following genes are shown: insulin receptor-beta subunit (*INSRβ*), insulin receptor substrate 1 (*IRS1*), glucose transporter 4 (*GLUT4*), carnitine palmitoyltransferase I (*CPT1*), uncoupling protein 3 (*UCP3*), and peroxisome proliferator-activated receptor alpha (*PPARα*). Panel A, data of 6-months old lambs; panel B, data of 2-years old sheep. These genes were all significantly up-regulated by the HCHF diet in 6-months old lambs, but only *INSRβ*, *IRS1*, and *GLUT4 we*re significantly up-regulated by the HCHF diet in 2-years old sheep. Data are logarithm transformed for statistical analysis, back-transformed, and expressed as least square mean ± standard error of the mean. NORM/CONV, NORM/HCHF, LOW/CONV, LOW/HCHF refer to experimental treatment groups. NORM and LOW refer to the prenatal nutrition offered to the twin-pregnant dams and fulfilling 100% and 50%, respectively, of daily requirements for energy and protein. CONV and HCHF refer to a moderate or high-carbohydrate-high-fat diet, respectively, fed during the first 6 months of postnatal life. Legends for both panels are shown at the top-right corner of panel A. “*” indicates significant differences between individual groups (*P*<0.05).

The postnatal diet was the main factor influencing mRNA expressions of target genes in skeletal muscle, in contrast to what was observed for protein expressions, and the glycolytic LD muscle was more responsive to the HCHF diet than the oxidative BF muscle. Thus, in LD, the HCHF diet enhanced expression of a range of genes involved in regulation of glucose uptake, insulin signalling and fatty acid oxidation: *INSRβ,* (*P = *0.06), *IRS1* (*P = *0.02), *GLUT4* (*P = *0.02), *CPT1* (*P<*0.0001), uncoupling protein 3 (*UCP3*, *P = *0.07), and peroxisome proliferator-activated receptor alpha (*PPARα*, *P = *0.04) (Figure 2A). The mRNA expressions of *INSRβ (P = 0.007), IRS1* (*P = *0.01), and *GLUT4* (*P = *0.05) continued to be higher in the adult HCHF compared to CONV sheep, even after 1½ years on the same moderate and (for HCHF sheep body-fat correcting) diet (Figure 2B).

**Figure 3:**
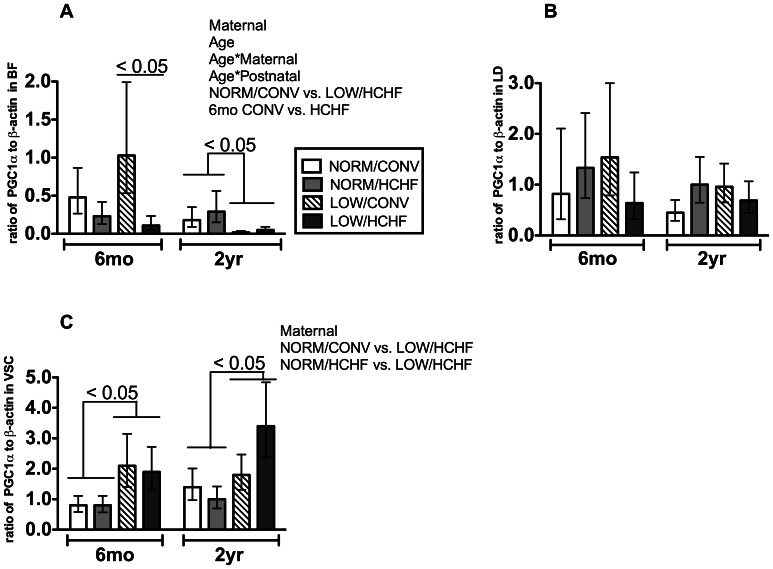
mRNA expressions of peroxisome proliferator activated receptor gamma coactivator 1 alpha isoform (*PGC1α*) in muscles. Panel A, *biceps femoris*; panel B, *longissimus dorsi*; panel C, *ventriculus sinister cordis*. Data are the ratio of target to beta-actin. Data are logarithm transformed for statistical analysis, back-transformed, and expressed as least square mean ± standard error of the mean. NORM/CONV, NORM/HCHF, LOW/CONV, LOW/HCHF refer to experimental treatment groups. NORM and LOW refer to the prenatal nutrition offered to the twin-pregnant dams and fulfilling 100% and 50%, respectively, of daily requirements for energy and protein. CONV and HCHF refer to a moderate or high-carbohydrate-high-fat diet, respectively, fed during the first 6 months of postnatal life. Experimental factors with P-values <0.05 are shown at the top-right corner for each graph. Legends for all panels are shown at the lower-right corner of panel A.

In BF, only 2 genes were affected by the postnatal diet. *CPT1* mRNA expression (*P = *0.03) was increased in 6-months old HCHF compared to CONV lambs (data not shown) just as seen in LD, whereas *PGC1α* mRNA expression (*P = *0.03) was depressed (Figure 3A). These postnatal diet induced changes did not persist into adulthood after diet correction. In BF, the prenatal diet affected expression of the *PGC1α*, but not of other genes, however this prenatal effect was only manifested in adulthood, where LOW sheep had markedly lower expressions compared to NORM sheep (*P = *0.005, Figure 3A).

Pre- and postnatal nutrition impacts on mRNA expression for the *PGC1α* gene were differentially expressed in the three different types of muscle as well as with age: expression was depressed in BF by the HCHF diet in lambs but not by the prenatal LOW nutrition exposure in adult ewes, there was no impact of pre- or postnatal nutrition at all on expression in LD (Figure 3B), and *PGC1α* mRNA expression was upregulated in VSC in both lambs and adult ewes with a history of late-gestation undernutrition (LOW) (P = 0.003, Figure 3C).

Pre-and postnatal nutrition did not affect mRNA expression for any other genes in VSC with the exception of *PPARδ* as described in the following.

**Figure 4:**
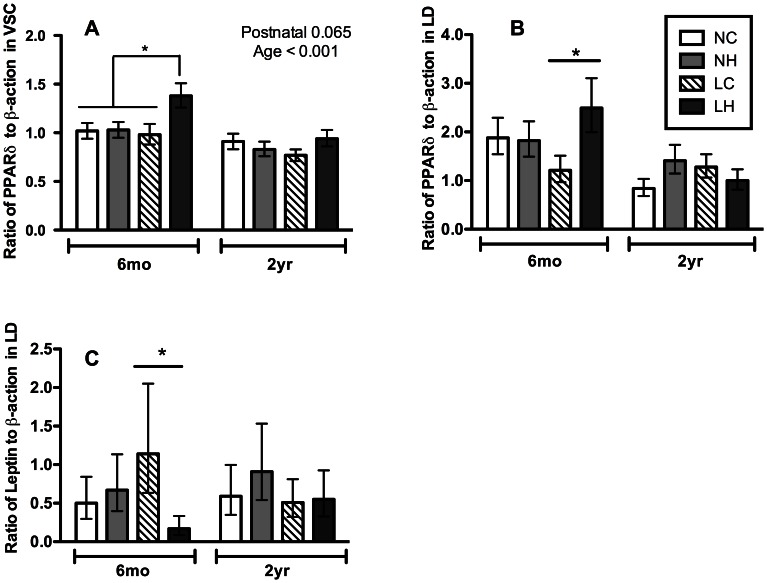
mRNA expressions that affected by the interaction of maternal LOW diet and postnatal HCHF diet. Panel A, peroxisome proliferator-activated receptor (*PPARl*) mRNA expression in *ventriculus sinister cordis* (VSC); panel B, *PPARC* mRNA expression in *longissimus dorsi* (LD); Panel C, leptin mRNA expression in LD. Data are the ratio of target to beta-actin. Data are logarithm transformed for statistical analysis, back-transformed, and expressed as least square mean ± standard error of the mean. NC, NH, LC, LH refer to experimental treatment groups. N and L refer to the prenatal nutrition offered to the twin-pregnant dams and fulfilling 100% and 50%, respectively, of daily requirements for energy and protein. C and H refer to a moderate or high-carbohydrate-high-fat diet, respectively, fed during the first 6 months of postnatal life. Experimental factors with P-values *<*0.05 are shown at the top-right corner for each graph. Legends for all panels are shown at the upper-right corner of panel B.

In the 6-months old lambs, postnatal diet induced changes in mRNA expression for two genes, peroxisome proliferator-activated receptor delta (*PPARδ*) and *leptin* (determined as a marker for intramuscular adipocytes in muscle) depended on the prenatal nutrition history (Figure 4). Thus, in LOW/HCHF lambs compared to lambs from other treatment groups, *PPARδ* mRNA expression was increased in the VSC muscle (LOW/HCHF vs. NORM/CONV, *P = *0.06; LOW/HCHF vs. NORM/HCHF, *P = *0.03; LOW/HCHF vs. LOW/CONV, *P* = 0.005) (Figure 4A) and in the LD muscle (*P = *0.03) (Figure 4B), whereas *leptin* mRNA expression was halved in LOW/HCHF as compared to LOW/CONV lambs (*P = *0.04) (Figure 4C).

The mRNA expressions of the peroxisome proliferator-activated receptor gamma (*PPARγ*), *PGC1β*, and fat mass and obesity associated protein (*FTO*) were unaffected by both the prenatal diet and the early postnatal diet in all the muscles studied (data not shown).

### Lipid Content and Fatty Acid Compositions of BF, LD, and VSC Muscles

No effect of the prenatal nutrition on muscle lipid content or lipid fatty acid compositions were observed in neither lambs nor adult sheep.

The HCHF diet increased total TG and Cer contents in all the three muscles of 6-months old lambs (all *P*<0.05) (Table 2). LOW/HCHF lambs accumulated more TG in BF muscle compared to the NORM/HCHF lambs (*P*<0.05). At 2-years of age, the total TG content in the BF muscle of HCHF sheep was still higher than that observed for the CONV sheep (P<0.05), whereas the total TG content in the LD and VSC muscle from HCHF sheep had returned to similar levels as that for CONV sheep (data not shown). In the 2-years old sheep, there was no difference in Cer content among the four groups for any of the three muscles (data not shown).

**Table 2:** Total triglyceride (TG) and ceramide (Cer) contents in muscles of 6-months old lambs.

	BF		LD		VSC	
	TG	Cer	TG	Cer	TG	Cer
NORM**/CONV**	16.0 (±6.66)	32.7 (±4.16)	12.6 (±6.87)	43.3 (±3.50)	3.9 (±3.28)	33. (±2.89)
LOW**/CONV**	9.3 (±0.96)	32.7 (±3.04)	12.5 (±6.76)	42.4 (±4.51)	4.8 (±4.60)	38.1 (±2.41)
NORM**/HCHF**	32.6 (±7.48)	42.4 (±4.11)	39.4 (±15.6)	54.9 (±5.36)	6.6 (±8.08)	52.8 (±3.26)
LOW**/HCHF**	46.5 (±6.34)	45.5 (±4.51)	50.0 (±15.4)	55.4 (±6.53)	9.2 (±3.22)	56.2 (±7.67)

The three muscles were: *biceps femoris* (BF), *longissimus dorsi* (LD), and *ventriculus sinister cordis* (VSC).

Data are expressed as means ± standard deviations. Unit for total TG content is mg per gram tissue (mg/g), and unit for total Cer content is nmol per gram tissue (nmol/g). Both TG and Cer contents for each of the muscles were significantly increased by the HCHF diet. NORM/CONV, NORM/HCHF, LOW/CONV, LOW/HCHF refer toexperimental treatment groups. NORM and LOW refer to the prenatal nutrition offered to the twin-pregnant dams and fulfilling 100% and 50%, respectively, of daily requirements for energy and protein. CONV and HCHF refer to a moderate or high-carbohydrate-high-fat diet, respectively, fed during the first 6 months of postnatal life.

**Figure 5:**
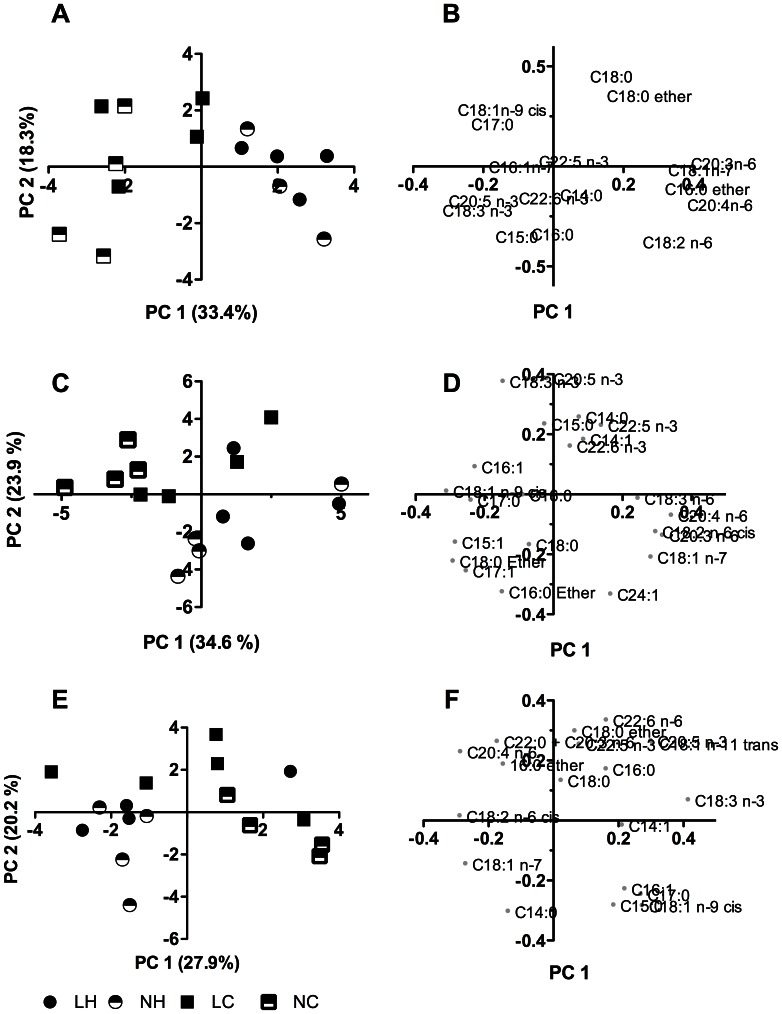
Principal component analysis score and loading plots for muscle phospholipid components of 2-years old sheep. Panel A and B, *biceps femoris*; panel C and D, *longissimus dorsi*; panel E and F, *ventriculus sinister cordis*. Panels A, C, and E are score plots; panels B, D, and F are loading plots. The first two principle components (PC) are plotted. NC, NH, LC, LH refer to experimental treatment groups. N and L refer to the prenatal nutrition offered to the twin-pregnant dams and fulfilling 100% and 50%, respectively, of daily requirements for energy and protein. C and H refer to a moderate or high-carbohydrate-high-fat diet, respectively, fed during the first 6 months of postnatal life. Legends for all panels are shown at the bottom of panel E.

Visualized by PCA, the HCHF diet as expected had substantial impact on the FA-composition in the PL fraction of each muscle type in 6-months old lambs (data not shown). Interestingly, after dietary correction and feeding with the same diet for 1.5 years, these remarkable differences in PL FA-composition were still evident in 2-years old sheep (Figure 5).

**Figure 6:**
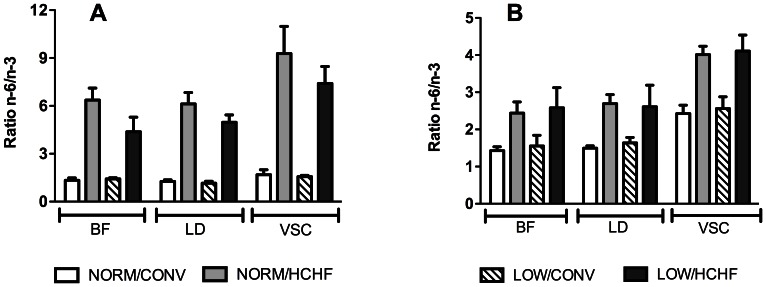
The ratio of omega-6/omega-3 (n-6/n-3) polyunsaturated fatty acids in the phospholipid fraction of muscles. Panel A, data of 6-months old lambs; panel B, data of 2-years old sheep. The three muscles were: *biceps femoris* (BF) *longissimus dorsi* (LD), and *ventriculus sinister cordis* (VSC). Data are expressed as means **±** standard deviations. NORM/CONV, NORM/HCHF, LOW/CONV, LOW/HCHF refer to experimental treatment groups. NORM and LOW refer to the prenatal nutrition offered to the twin-pregnant dams and fulfilling 100% and 50%, respectively, of daily requirements for energy and protein. CONV and HCHF refer to a moderate or high-carbohydrate-high-fat diet, respectively, fed during the first 6 months of postnatal life. Legends for all panels are shown at the bottom of the figure.

**Figure 7:**
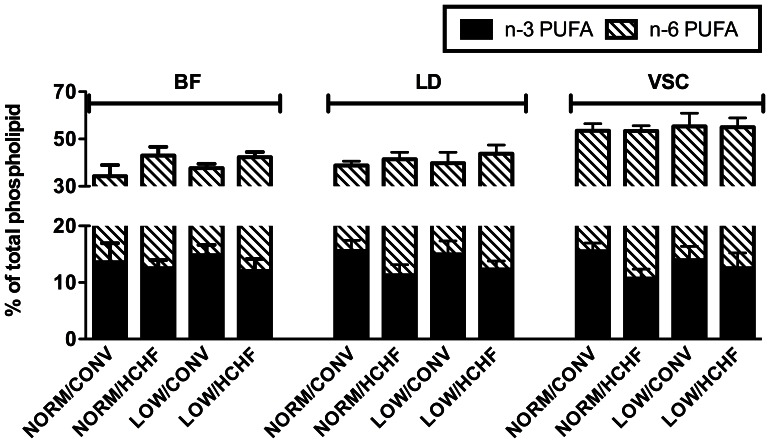
Phospholipid omega-6 and omega-3 polyunsaturated fatty acids (PUFA) contents of adult sheep. The percentile of omega-6 (n-6) or omega-3 (n-3) PUFA are shown for *biceps femori*s (BF), *longissimus dorsi* (LD), and *ventriculus sinister cordis* (VSC). Data are shown as means **±** standard deviations. NORM/CONV, NORM/HCHF, LOW/CONV, LOW/HCHF refer to experimental treatment groups. NORM and LOW refer to the prenatal nutrition offered to the twin-pregnant dams and fulfilling 100% and 50%, respectively, of daily requirements for energy and protein. CONV and HCHF refer to a moderate or high-carbohydrate-high-fat diet, respectively, fed during the first 6 months of postnatal life. Legends are shown at the upper-right corner of the figure.

For TG and PL FA-compositions, the HCHF diet caused a pronounced increase in the omega-6 polyunsaturated fatty acid (n-6 PUFA) content that was associated with decreased n-3 PUFA content in TG and PL. This led to substantially higher n-6/n-3 PUFA ratios in the three muscle types in the HCHF lambs (Figure 6A), while the total PL PUFA content in lambs and sheep were unaffected by the HCHF diet. The impacts of the HCHF diet on n-6 and n-3 PUFA contents were seen to different extents in the adult sheep (Figure 7). The 2-years old HCHF sheep continued to have both higher n-6 PUFA and lower n-3 PUFA contents in the skeletal muscles BF and LD, and therefore higher n-6/n3 PUFA ratio in the PL fraction compared to that of CONV sheep (Figure 6B). The cardiac VSC muscle of the 2-years old HCHF sheep showed no significant reduction in n-6 PUFA content in the PL fraction, whereas the VSC muscle of adult HCHF sheep had lower n-3 PUFA content compared to that of the CONV sheep. Therefore, the cardiac VSC muscle of 2-years old HCHF sheep also had an elevated n-6/n-3 PUFA ratio as observed in BF and LD muscles (Figure 6B).

### Protein-protein Interaction Networks Analyses

**Figure 8:**
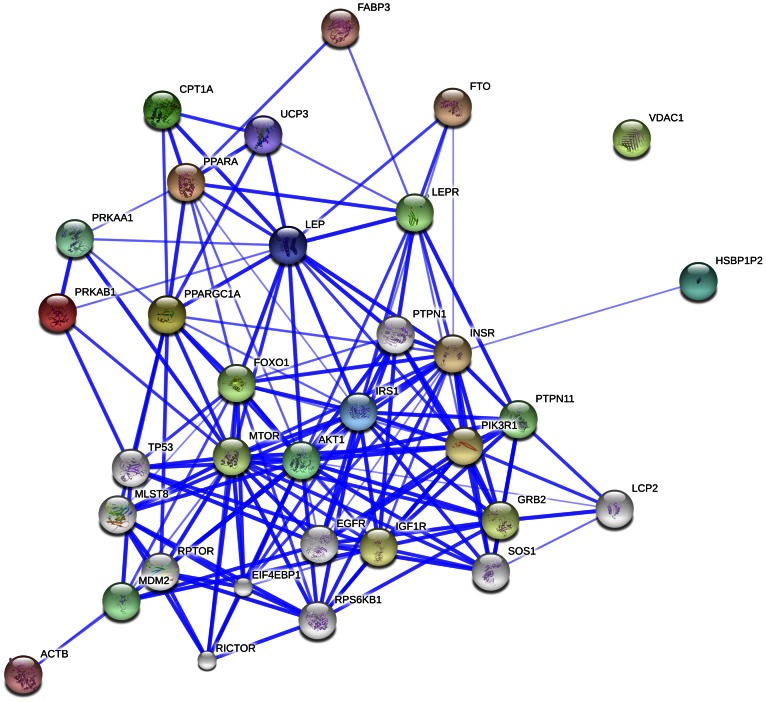
Confidence view of protein-protein interaction network. The thickness of the blue line between two nodes indicates the confident level of the association of these two nodes. The thicker the blue line is, the higher the confidence level that the two nodes are associated with each other.

**Figure 9:**
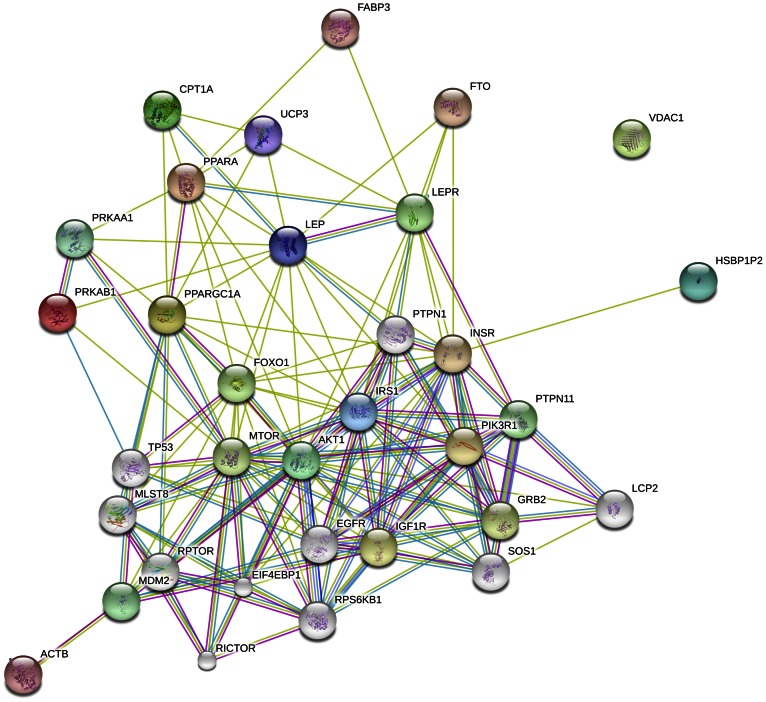
Evidence view of protein-protein interaction network. A red line indicates the presence of fusion evidence; a green line - neighbourhood evidence; a blue line - co-occurrence evidence; a purple line - experimental evidence; a yellow line – textmining evidence; a light blue line - database evidence.

We used STRING to investigate interactions of our target genes that are known to be involved in muscle energy metabolism, similarly to how it was reported by Kadarmideen and Janss [33] for stress related candidate genes in pigs, and by Kadarmideen [34] for genes involved in mammalian reproduction. The STRING analyses revealed many (some 30) other candidate genes that are likely to be involved in the fetal and postnatal nutrition programming of insulin signalling and energy metabolism in muscle. The actual results of PPI networks are given in two formats: a network with varying levels of confidence (Figure 8) and a network showing various evidences that support interactions (Figure 9). The input nodes are coloured and nodes of a higher iteration/depth are in white. Each family of proteins is assigned a different colour.

In the confidence view of the network (Figure 8), the confidence score for each interaction is the approximate probability that a predicted link exists between two nodes in the same metabolic map in the KEGG database. The thicker the line, the higher the confidence. In the evidence network (Figure 9), the edges, i.e. predicted functional links, consist of up to seven lines: one colour for each type of evidence. A red line indicates the presence of fusion evidence; a green line - neighbourhood evidence; a blue line - co-occurrence evidence; a purple line - experimental evidence; a yellow line – text mining evidence; a light blue line - database evidence; a black line - coexpression evidence. In the original interactive network, it is possible to hover over an edge that will display the combined association score, and clicking on it can give the detailed evidence breakdown. From the STRING gene/protein network analyses, it is clear that more molecular markers very strongly interact with the target genes/markers we studied here and it includes, for example, Insulin-like growth factor 1 receptor (*IGF1R*), forkhead box protein O1 (*FOXO1*), growth factor receptor-bound protein 2 (*GRB2*), lymphocyte cytosolic protein 2 (*LCP2*), son of sevenless homolog 1 (*SOS1*), target of rapamycin complex subunit LST8 (*MLST8*) and mammalian target of rapamycin (*MTOR*). It is recommended that future studies include these target genes to validate their effects in sheep and other animal models.

## Discussion

In the present study, we aimed to investigate whether the previously observed phenotypic alterations of glucose-insulin axis function (i.e. insulin sensitivity, glucose tolerance, and pancreatic insulin secretion) by pre- and postnatal nutritional insults in sheep [22] can be ascribed to metabolic programming of muscle functions. We have previously reported results from *in vivo* studies of glucose-insulin axis function in the experimental animals, which are published elsewhere [24], and in interpretation of these results we took into careful consideration that gender effects could only be evaluated in adolescent lambs, where both females and males were studied. Furthermore, age effects could only be evaluated in female animals, due to the experimental design. For the results reported in the present paper on muscle specific traits for molecular metabolism and lipid composition, it must be born in mind that we are not able to separate the effects of gender and ageing, since lambs slaughtered at 6-months of age were predominantly males, and the 2-years old adult sheep were exclusively females. Even though we measured only a selection of markers reported to be well-correlated with insulin sensitivity in LBW human and rodent subjects, we considered that other markers could potentially also contribute to the development of muscle insulin resistance. This idea was subsequently supported by the PPI network analyses of our targets. Thus, the interfering effect of fetal programing could probably disturb the protein-protein interactions between our targets and other candidate genes. With these considerations in mind, our findings suggest that exposure to prenatal undernutrition had only marginal effects on key markers of muscle energy metabolism. Thus, the exposure to a prenatal LOW level of nutrition affected insulin signalling molecules only in muscles of lambs, but not in adult sheep, although signs of insulin resistance were clearly revealed only in the adult sheep and not in the lambs, as previously reported [24]. Muscle phospholipid fatty acid composition reflected dietary fatty acid composition from the feeds provided during the early postnatal life. These compositional changes on structural lipids could, however, not be related to the expressional changes of key markers of muscle energy metabolism, nor could they be readily related to the observed changes in whole-body insulin sensitivity, which in adults were affected by pre- and not postnatal nutrition as previously mentioned. The muscle type was found to determine the nature of metabolic adaptations to nutritional exposures during the late-gestation and early-postnatal life.

### Late-gestation under-nutrition had only Marginal Effects on Key Markers of Muscle Energy Metabolism, but did Affect Insulin Signalling Proteins

The prenatal LOW nutrition exposure permanently reduced *GLUT4* protein in the BF muscle of the young sheep progenies, which agrees with observations from the maternal low-protein rat model and low-birth weight young men, where skeletal muscle *GLUT4* protein expression were also depressed by prenatal nutrition [10]. The contribution of this single muscle or the oxidative skeletal muscle type to whole-body insulin sensitivity and glucose clearance in sheep is not known in quantitative terms, but our observations indicate that sheep oxidative skeletal muscle can developed signs of glucose transportation deficiency prior to significant changes in whole-body insulin and glucose homeostasis.

To our surprise, our results did not support the existence of a link between the late-gestation LOW level of nutrition and development of overall muscle metabolic dysfunctions and subsequently whole-body insulin insensitivity. In human and rat LBW individuals, such a link has been found, and observations have suggested a link to altered expressions of muscle insulin signalling molecules and *PGC1α* [10], [12], [35]. Although protein expressions of insulin signalling molecules were reduced in muscles of LOW lambs, these changes were not evident in the young adult sheep, except for *GLUT4*, but it was only in these young adult sheep that signs of whole-body insulin resistance were clearly detected [22]. The *PGC1α* mRNA expression in different muscle types was differentially affected by pre- and postnatal nutrition exposures, and only in VSC did the altered expressions persist into adulthood. We are therefore unable to establish any cause-and-effect relationship between prenatal undernutrition and later development of muscle insulin insensitivity in the present study.

In the rat LBW model, nutritional insults are normally introduced very early in gestation and persist throughout the whole gestation period. However, the secondary myogenesis is not concluded during gestation but continues into the lactation period in rat pups, whereas it is terminated before entering the late-gestation period in sheep and humans [19], [20], [21]. Recent human studies have suggested that fetal growth during late-gestation is not a major determinant, nor associated with the risk, of type II diabetes, but such associations could be established to fetal growth prior to the third trimester [7], [36]. Thus, as we apply maternal nutrition restriction only during the last six weeks of gestation which is equivalent to the last trimester of pregnancy, the lack of any major effect of the late-gestation LOW nutrition exposure in our sheep model does not necessarily contradict observations from the rat or the human LBW model. Instead, our observations indicate that the time-window of exposure may determine the extent to which fetal programming impact on postnatal muscle functions, and muscle development and maturation may not be particularly vulnerable to nutritional insults during late fetal life.

### High-carbohydrate-high-fat Diet, Phospholipid Fatty Acid Composition, and Muscle Insulin Signalling

Fatty acids are important components of cells, and PL FA-composition is especially critical for the structure and function of cell membranes [37]. It is well-known that dietary supply of polyunsaturated fatty acids directly can influence the FA-composition in skeletal and cardiac muscles [38], [39]. Thus, high dietary supply of one kind of polyunsaturated fatty acid (n-3 or n-6) can specifically increase the muscle content of this PUFA-type at the expense of the other type. The major PUFA source of the CONV lamb diet was hay, whereas the major PUFA source of the HCHF lamb diet was maize. Both hay and maize have high n-6 PUFA contents (hay, approximately 60 g linoleic acid per 100 g fat; maize, approximately 61 g linoleic acid per 100 g fat), but hay has higher n-3 PUFA content than maize (hay, approximately 16g α-linolenic acid per 100g fat; maize, approximately 0.7 g α-linolenic acid per 100g fat) [40]. Thus, for our 6-months old HCHF lambs, the changes in muscle TG and PL FA-compositions, especially the changes on n-6/n-3 PUFA ratio, were expected due to the FA-composition of the early postnatal diet.

It was surprising, however, that the PL FA-compositions of adult sheep skeletal muscles continued to reflect the differences introduced by the lamb diet, even though all the sheep had been fed on the same identical (and for HCHF sheep body fat correcting) diet for 1.5 years. The most obvious explanation for this long-term effect is the extremely low turnover of phospholipid PUFA and hence delayed ‘washing-out’ of previously incorporated fatty acids in skeletal muscle cell membranes. To the best of our knowledge, the PUFA turnover rate in the skeletal muscle PL fraction has never been reported in humans or sheep. Our observations suggest that the PUFA half-life in the sheep skeletal muscles’ PL faction might be as long as 1.5 years. The fact that the n-6 PUFA content in VSC muscle was identical among the four groups, while the n-3 PUFA content was still reduced in the HCHF individuals at 2-years of age, showed that PUFA composition in cell membranes is regulated differently in cardiac muscle than in the skeletal muscles. Further investigations are required to clarify whether alterations of muscle PUFA metabolism priorities, which subsequently influences FA-composition of muscle lipids, could be a possible target of programming by early-life nutrition.

The link between long-chained PUFA composition in skeletal muscle PL and whole-body insulin sensitivity, which is suggested by human studies, might be coinciding with obesity induced whole-body insulin resistance [17], [18], [41]. In our study, muscle n-6/n-3 PUFA ratio was strongly increased in HCHF lambs, but irrespective of this, peripheral insulin sensitivity was increased in these lambs as compared to CONV fed lambs, as they were able to maintain normal glucose tolerance despite a markedly reduced ability to secrete insulin in response to an intravenous glucose challenge [24]. A similar uncoupling of insulin sensitivity from FA composition in muscle PL’s was observed in the young adult sheep, where the early-life HCHF diet still affected muscle PL FA-composition, but the impact of the postnatal diet on whole-body insulin sensitivity and glucose tolerance had disappeared in the adult HCHF sheep after 1½ years on a body-fat correcting diet. Thus, the n-6/n-3 PUFA ratio disagreed with changes in glucose-insulin axis function in our sheep model, regardless of the degree of fatness of the individuals. We do not rule out that the observed alterations of muscle PL FA-composition can affect other aspects of muscle function like contractility [42], [43], but our studies do not support that pre- and postnatal induced changes in PL FA composition should be major determinants of skeletal muscle insulin signalling or sensitivity later in life.

### Role of Muscle Type on Metabolic Adaptations to Nutrition

The three muscle types responded differentially to nutritional insults. As we expected, the expressions of energy metabolism related genes for cardiac muscle were in general quite resistant to pre- and especially postnatal nutritional insults. This observation agrees with reports from another sheep model, where the cardiac muscle energy metabolism related gene expression pattern was unaffected by early-mid gestation nutrition restriction [24].

Previous reports have proposed that the oxidative skeletal muscle types may be more sensitive with respect to exposures leading to intrauterine growth restriction [26], [44], [45] or towards postnatal high-fat feeding [25], [46], [47] compared to the glycolytic skeletal muscle types. We did observe that the BF muscle had a greater ability than the other muscles to accumulate TG upon exposure to the postnatal HCHF diet, and BF was the only muscle in HCHF sheep that continued to have a high TG content after dietary (and body-fat) correction. This could indicate that the plasticity of fatty acid metabolism in the oxidative muscle (type-I fibre rich) BF is more prone towards perinatal and postnatal nutritional programming than the glycolytic LD (type-II fibre rich) and the cardiac VSC muscles. But there is no doubt that we in our studies observed the most dramatic effects of late gestation nutrition on protein expression, and of early postnatal nutrition on expression of mRNA, for a wide range of targets in the glycolytic LD muscle (Figures 1 and 2). We have no definite explanation for this discrepancy compared to earlier studies, but different metabolic properties of sampled muscles might explain differential observations in our sheep study compared to the above-mentioned rodent studies. Muscle biopsies are typically sampled from leg and arm muscles in human studies, and from leg muscles in rodent studies, and the LD muscle is rarely if ever sampled in species other than farm animals such as sheep, cattle and pigs.

Late-gestation undernutrition caused differential *PGC1α* mRNA expression changes in the three investigated muscle types (Figure 3). The *PGC1α* gene is one of the key regulatory factors that coordinates mitochondrial biogenesis and maintains the mitochondrial network [48], and reduced skeletal muscle *PGC-1α* expression is believed to be associated with impaired whole-body insulin sensitivity [11]. Our observations on *PGC1α* suggest that the adaptations of muscle oxidative metabolism upon exposure to a prenatal LOW level of nutrition are muscle type-dependent, and these differential adaptations may be associated with specific metabolic properties of the muscle and prenatal blood-flow re-partitioning [49], [50], [51].

### Conclusions

We conclude that early postnatal, but not late-gestation, nutrition had long-term consequences for glucose, insulin and fat oxidation markers in LD and for lipidomes of all the studied muscles. Muscle did therefore apparently not contribute significantly to the previously reported development of adult whole-body insulin insensitivity induced by late-gestation under-nutrition in our sheep model. Other tissues or organs must clearly be responsible for this. Systems biology analyses revealed more molecular markers e.g. (*GRB2*, *FOXO1*, *IGF1R*) that very strongly interacted with the target genes we investigated. Future studies should include these target genes to validate their effects in sheep as compared to other experimental models and humans. Further investigations are required to elucidate the role played by the muscle lipidome on long-term metabolic programming of muscle development and function. The results add to the mounting evidence of quite diverse metabolic adaptability among different muscle types.

## Supporting Information

Information S1 **Experimental design of the Copenhagen sheep model.** NORM/CONV, NORM/HCHF, LOW/CONV, LOW/HCHF refer to experimental treatment groups. NORM and LOW refer to the prenatal nutrition offered to the twin-pregnant dams and fulfilling 100% and 50%, respectively, of daily requirements for energy and protein. CONV and HCHF refer to a moderate or high-carbohydrate-high-fat diet, respectively, fed during the first 6 months of postnatal life.
(TIFF)

Information S2 **β-tubulin cannot serve as loading control of western blotting.** One of the gels and its associated membrane are used as an example. The colour intensities of coomassie staining, Ponceau S staining, and β-tubulin protein expression are shown in the top graph. Data are shown as the ratio of sample value to the mean value of the muscle standards (stand.). The lanes on gel/membrane are aligned to the sample name shown on the top graph. Legend and coefficient of variance (CV) are given at the top-right corner.
(TIFF)

Information S3 **Quality and specificity of western blotting of** ***biceps femoris*** **.** Target proteins are insulin receptor-beta subunit (*INSRe*), protein kinase zeta (*PKCζ*), and glucose transporter 4 (*GLUT4*). The data shown here are derived from the same membrane.
(TIFF)

Information S4 **Quality and specificity of western blotting of** ***longissimus dorsi*** **.** Target proteins are insulin receptor-beta subunit (*INSRβ*), protein kinase zeta (*PKCζ*), and glucose transporter 4 (*GLUT4*). The data shown here are derived from the same membrane.
(TIFF)

Information S5 **Quality and specificity of western blotting of** ***ventriculus sinister cordis*** **.** Target proteins are insulin receptor-beta subunit (*INSRβ*), protein kinase zeta (*PKCζ*), and glucose transporter 4 (*GLUT4*). The data shown here are derived from the same membrane.
(TIFF)

Information S6 **Quality and specificity of western blotting of** ***ventriculus sinister cordis*** **.** Target proteins are phosphoinositide 3 kinase-p85 regulatory subunit (*PI3K-p85*) and phosphoinositide 3 kinase-p110 catabolic subunit (*PI3K-p110*). The data shown here are derived from the same membrane.
(TIFF)
