# Supplementary figures and images for: Pre- and Early-Postnatal Nutrition Modify Gene and Protein Expressions of Muscle Energy Metabolism Markers and Phospholipid Fatty Acid Composition in a Muscle Type Specific Manner in Sheep
Source: PLoS One. 2013 Jun 6;8(6):e65452. doi: 10.1371/journal.pone.0065452 (PMC3675032; doi:10.1371/journal.pone.0065452)

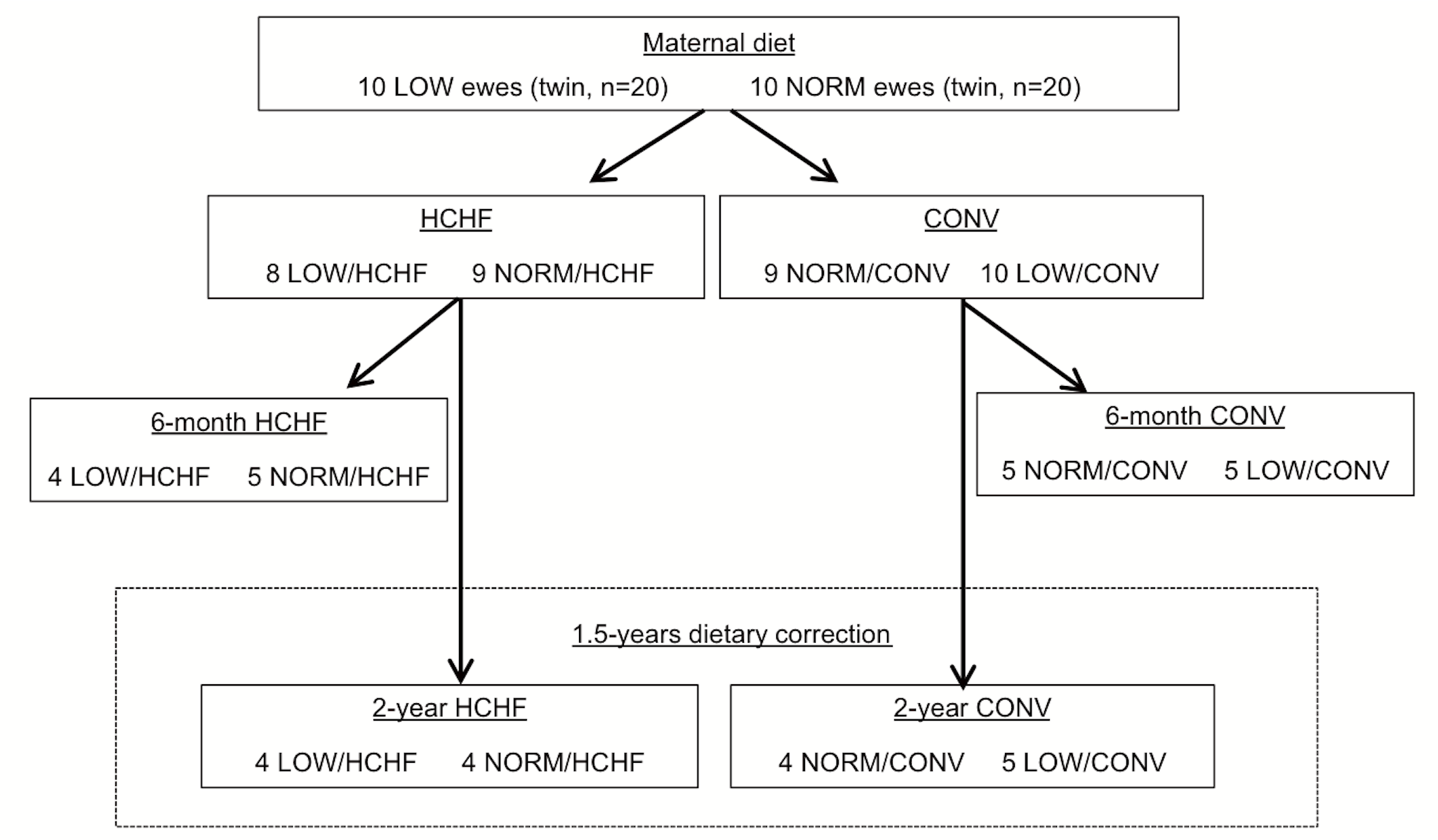

Supplement: Information S1 — Experimental design of the Copenhagen sheep model. NORM/CONV, NORM/HCHF, LOW/CONV, LOW/HCHF refer to experimental treatment groups. NORM and LOW refer to the prenatal nutrition offered to the twin-pregnant dams and fulfilling 100% and 50%, respectively, of daily requirements for energy and protein. CONV and HCHF refer to a moderate or high-carbohydrate-high-fat diet, respectively, fed during the first 6 months of postnatal life. (TIFF) [file pone.0065452.s001.tif]

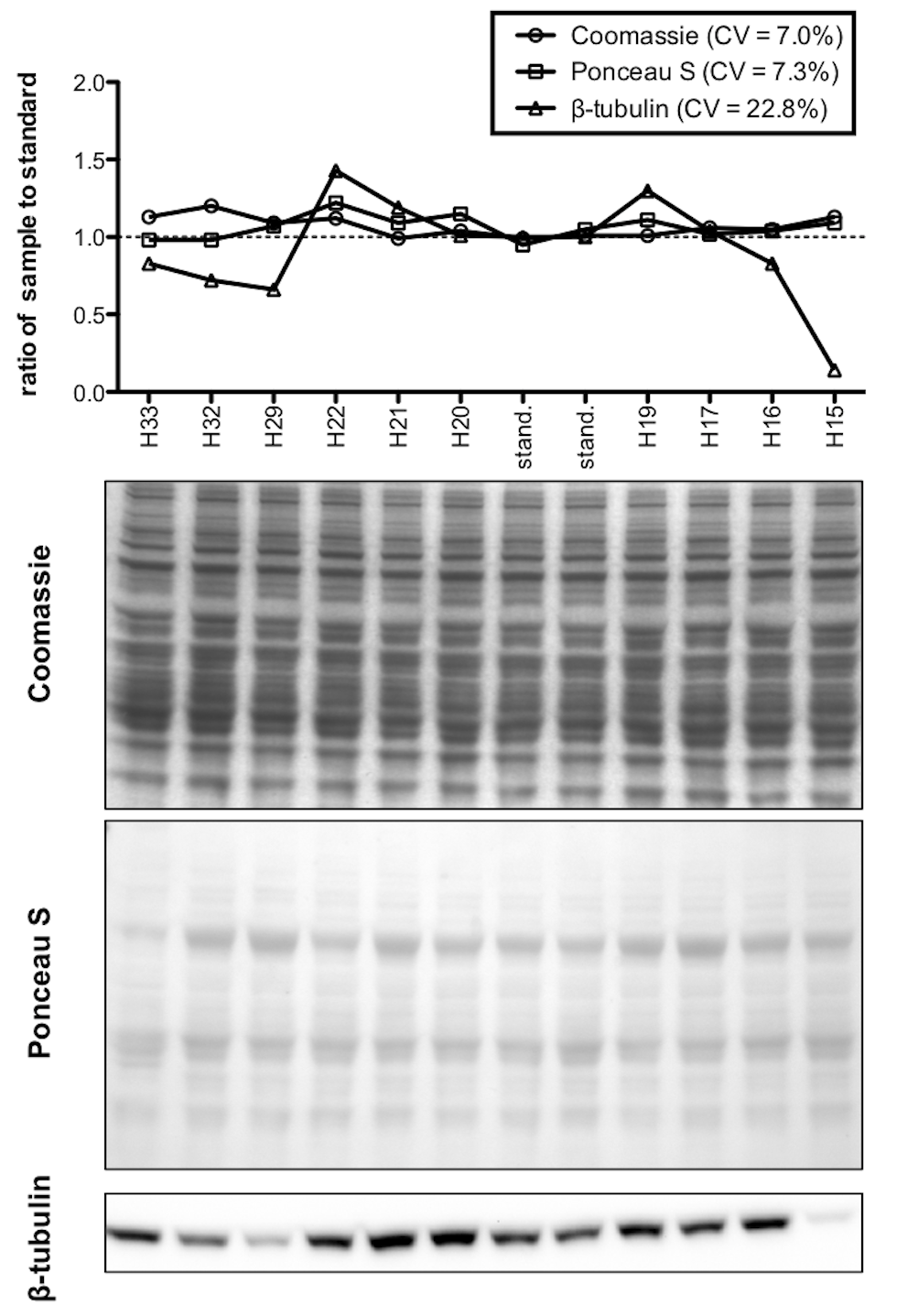

Supplement: Information S2 — β-tubulin cannot serve as loading control of western blotting. One of the gels and its associated membrane are used as an example. The colour intensities of coomassie staining, Ponceau S staining, and β-tubulin protein expression are shown in the top graph. Data are shown as the ratio of sample value to the mean value of the muscle standards (stand.). The lanes on gel/membrane are aligned to the sample name shown on the top graph. Legend and coefficient of variance (CV) are given at the top-right corner. (TIFF) [file pone.0065452.s002.tif]

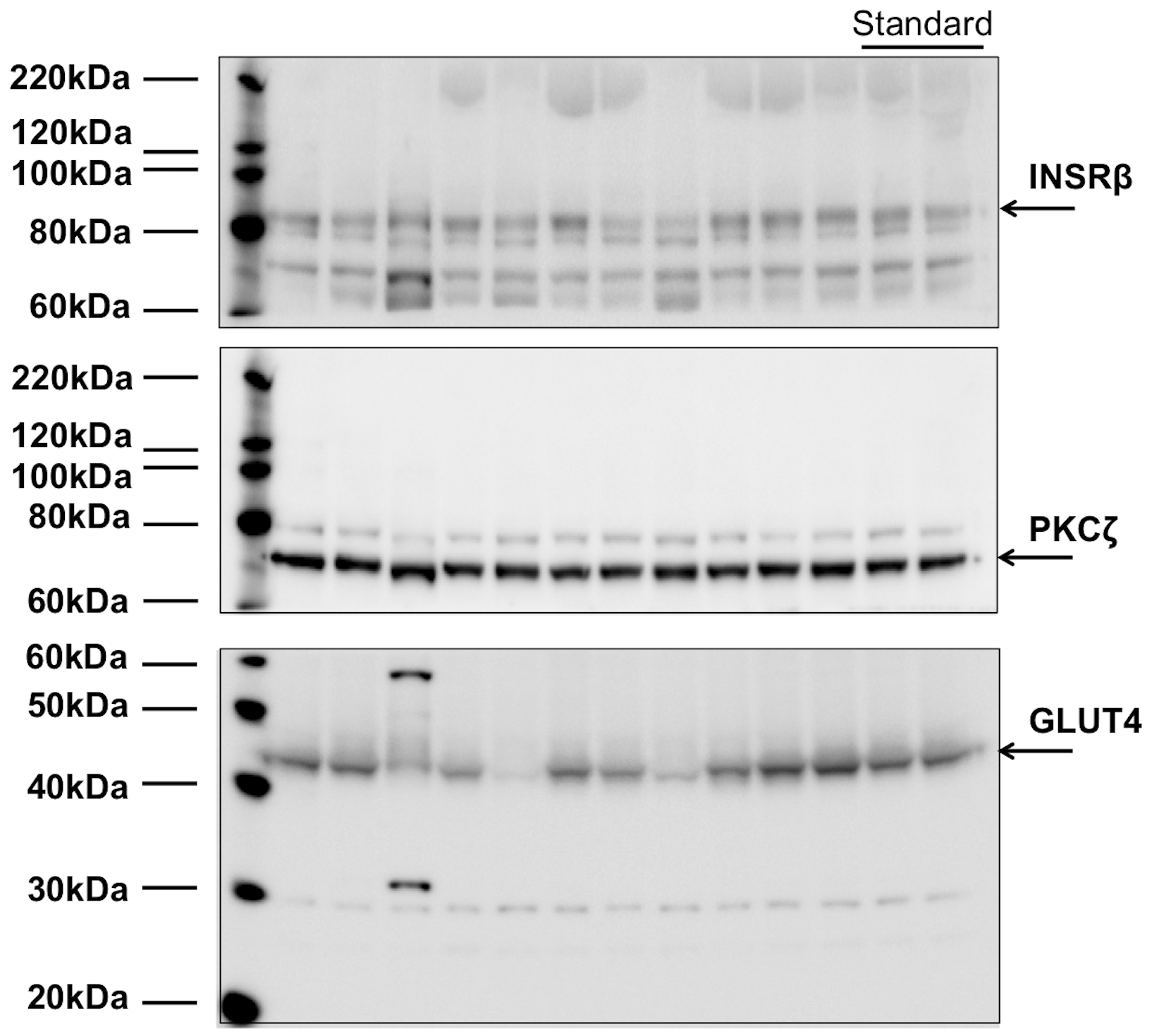

Supplement: Information S3 — Quality and specificity of western blotting of biceps femoris . Target proteins are insulin receptor-beta subunit (INSRe), protein kinase zeta (PKCζ), and glucose transporter 4 (GLUT4). The data shown here are derived from the same membrane. (TIFF) [file pone.0065452.s003.tif]

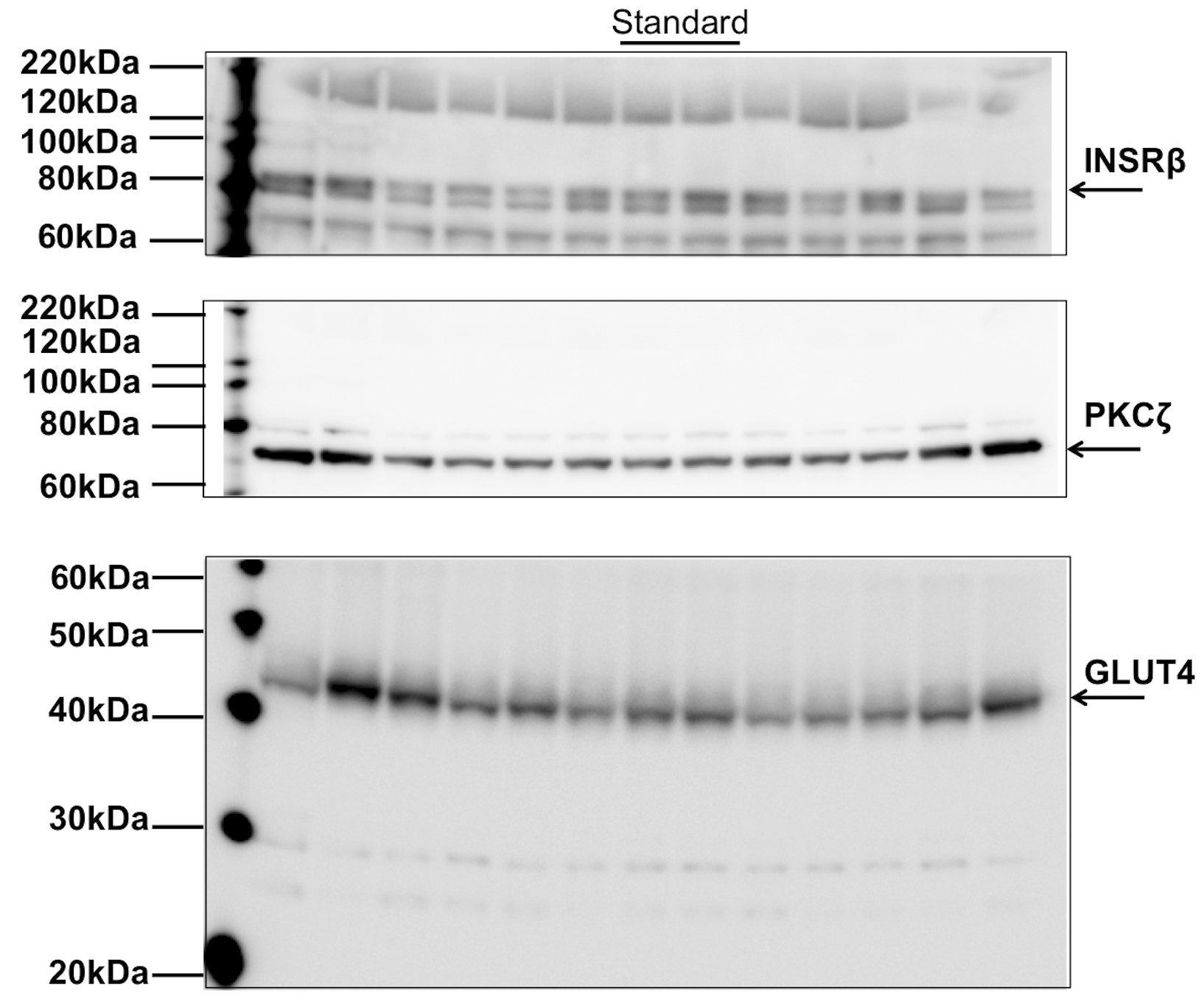

Supplement: Information S4 — Quality and specificity of western blotting of longissimus dorsi . Target proteins are insulin receptor-beta subunit (INSRβ), protein kinase zeta (PKCζ), and glucose transporter 4 (GLUT4). The data shown here are derived from the same membrane. (TIFF) [file pone.0065452.s004.tif]

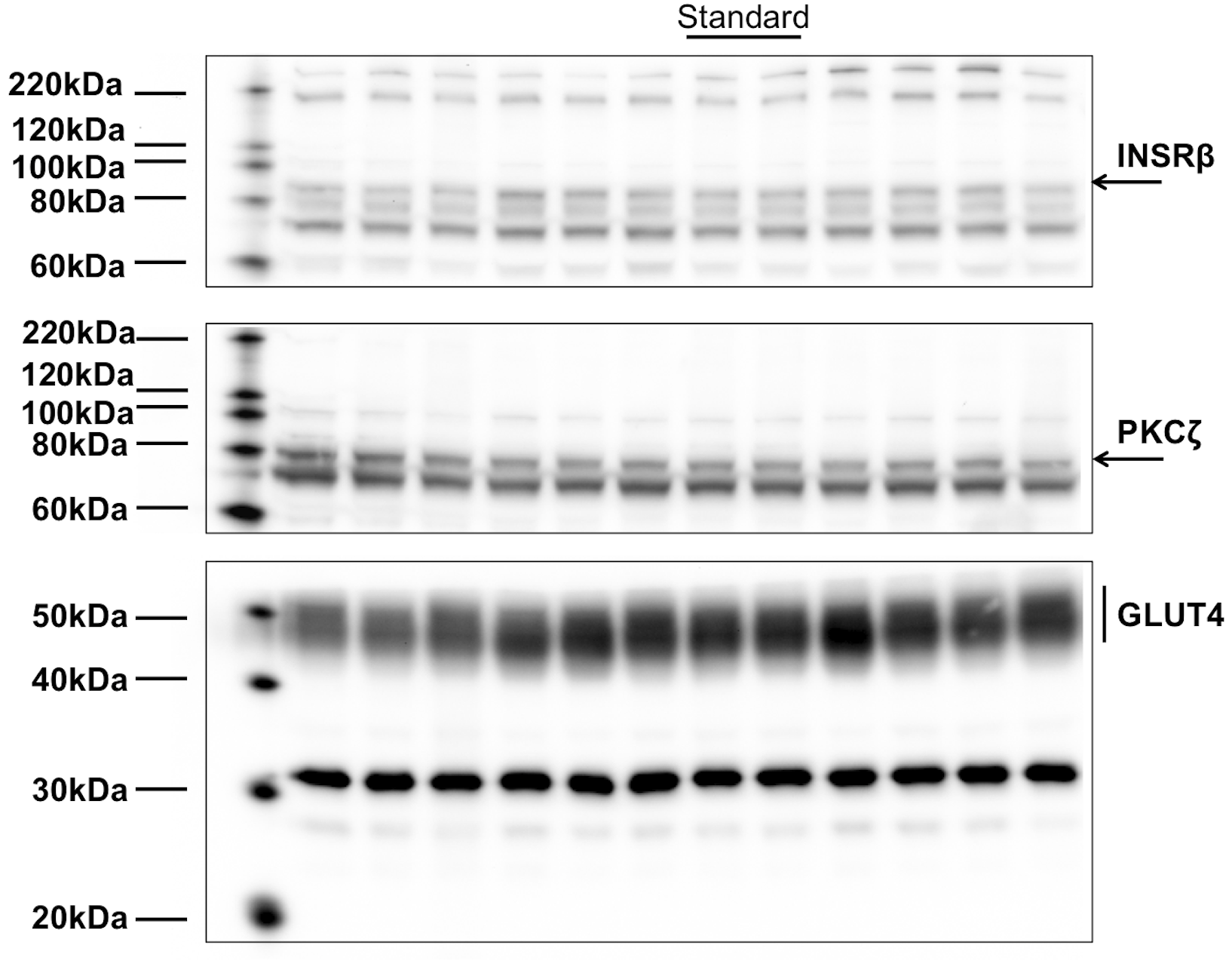

Supplement: Information S5 — Quality and specificity of western blotting of ventriculus sinister cordis . Target proteins are insulin receptor-beta subunit (INSRβ), protein kinase zeta (PKCζ), and glucose transporter 4 (GLUT4). The data shown here are derived from the same membrane. (TIFF) [file pone.0065452.s005.tif]

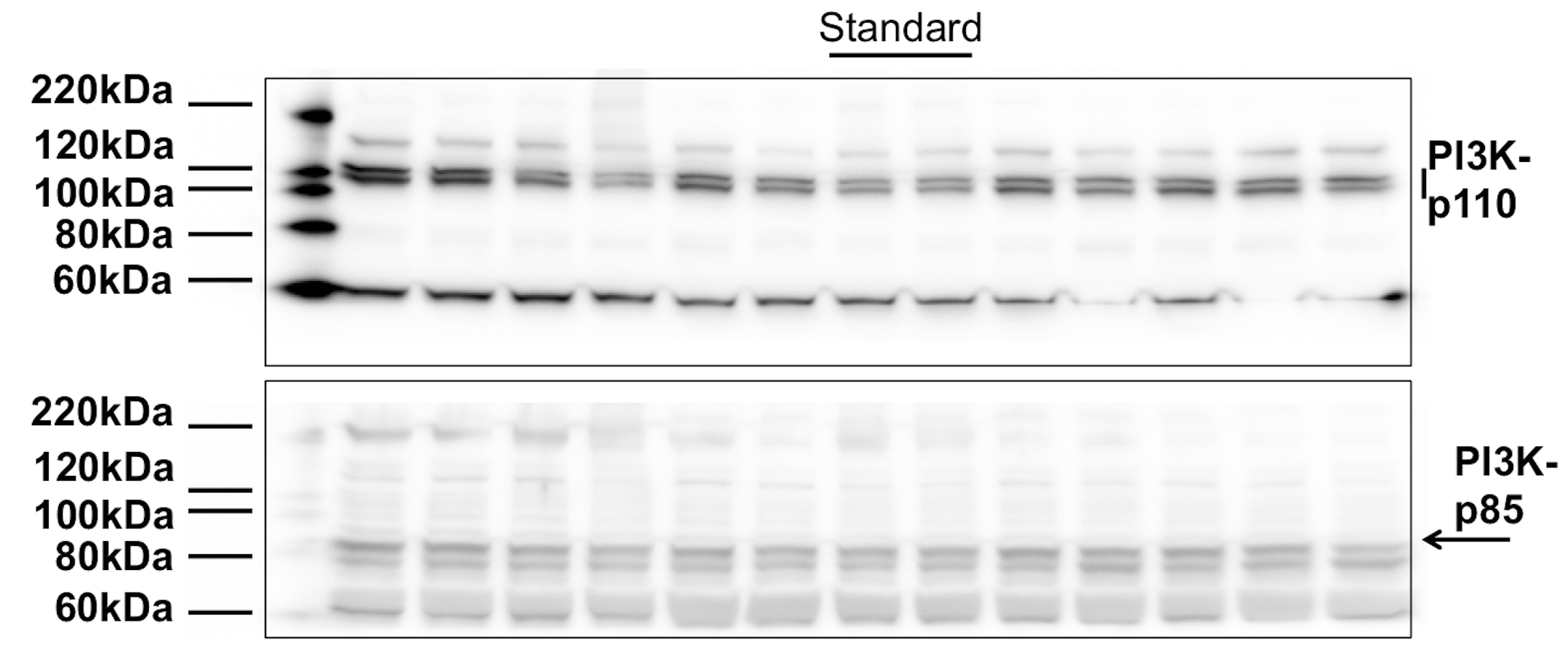

Supplement: Information S6 — Quality and specificity of western blotting of ventriculus sinister cordis . Target proteins are phosphoinositide 3 kinase-p85 regulatory subunit (PI3K-p85) and phosphoinositide 3 kinase-p110 catabolic subunit (PI3K-p110). The data shown here are derived from the same membrane. (TIFF) [file pone.0065452.s006.tif]
